# Integrated real-time imaging of executioner caspase dynamics, apoptosis-induced proliferation, and immunogenic cell death using a stable fluorescent reporter platform

**DOI:** 10.1038/s41420-025-02662-y

**Published:** 2025-08-06

**Authors:** Selen Selcen, Lena Wieland, Tiago De Oliveira, Michael Ghadimi, Lena-Christin Conradi, Günter Schneider, Leonie Witte, Matthias Wirth

**Affiliations:** 1 Department of General, Visceral and Pediatric Surgery, University Medical Center Göttingen, 37075 Göttingen, Germany; 2 Clinical Research Unit 5002, KFO5002, University Medical Center Göttingen, 37075 Göttingen, Germany; 3 CCC-N (Comprehensive Cancer Center Lower Saxony), 37075 Göttingen, Germany; 4 Department of Hematology, Oncology and Cancer Immunology, Charité - Universitätsmedizin Berlin, corporate member of Freie Universität Berlin and Humboldt-Universität zu Berlin, 12203 Berlin, Germany; 5 Max-Delbrück-Center for Molecular Medicine, 13125 Berlin, Germany; 6 German Cancer Consortium (DKTK), German Cancer Research Center (DKFZ), 69120 Heidelberg, Germany

**Keywords:** Cancer imaging, Preclinical research, Expression systems

## Abstract

Regulated cell death plays a central role in tissue homeostasis, disease progression, and therapeutic responses. However, tools to study these processes with high spatiotemporal resolution in physiologically relevant systems remain limited. Here, we present a fluorescent reporter cell system that enables real-time visualization of caspase-3/-7 activity via a DEVD-based biosensor, alongside a constitutive fluorescent marker for assessing successful transduction and cell presence. We generated stable cell lines expressing this reporter and adapted them to both 2D and 3D culture systems, including organoids. This platform allowed dynamic tracking of apoptotic events and viability loss at single-cell resolution. Using a proliferation dye, we also detected apoptosis-induced proliferation in neighboring cells. Furthermore, the system enabled simultaneous detection of immunogenic cell death via an endpoint measurement of surface calreticulin exposure by flow cytometry, supporting its application in studying immunogenic signaling. By measuring and integrating multiple cell death readouts by live-cell imaging, our system is well-suited for high-content screening and mechanistic dissection of different modes of cell death. When combined with complementary markers of pyroptosis and necroptosis, this platform may also be extended to investigate more complex, integrated forms of cell death.

## Introduction

Regulated forms of cell death, including apoptosis, are essential for maintaining tissue integrity, eliminating damaged cells, and orchestrating immune homeostasis. Apoptosis is primarily executed by a family of cysteine-aspartic proteases, the caspases, with caspase-3 and caspase-7 acting as key effector enzymes [1–3]. Activation of these executioner caspases triggers the systematic cleavage of structural and regulatory proteins, culminating in the organized dismantling of the dying cell [4].

While apoptosis has historically been viewed as immunologically silent, it is now recognized that certain forms of cell death can acquire immunogenic features, bridging innate and adaptive immune responses [5]. A fundamental challenge in cell death research lies in dynamically capturing the kinetics and molecular hallmarks of apoptosis and immunogenic cell death (ICD) with high spatiotemporal precision, especially within 3D culture systems that better recapitulate in vivo physiology [6]. ICD is a form of regulated cell death that stimulates adaptive immunity through the release of damage-associated molecular patterns (DAMPs) [7]. A key event in ICD is the surface exposure of calreticulin (CALR), which acts as an “eat me” signal promoting dendritic cell and macrophage uptake and antigen presentation [8]. For ICD to occur, dying cells must display sufficient antigenicity, release immunostimulatory signals (adjuvanticity), and exist within a permissive microenvironment [9]. ICD is a critical mechanism by which certain anticancer therapies enhance immune-mediated tumour clearance [10].

Traditional methods for detecting apoptosis - including Annexin V binding, caspase substrate cleavage, or TUNEL staining - largely rely on endpoint analyses and fail to accurately reflect the dynamic, asynchronous nature of apoptosis at the single-cell level [11]. In 2D cultures, these methods offer limited temporal resolution and hinder continuous tracking of cell fate, with imaging in 3D models further complicated by poor dye penetration, photobleaching, and signal heterogeneity [12].

To overcome these limitations, we developed a lentiviral-based, stable reporter system. A key feature of our platform is the utilization of the ZipGFP-based (green fluorescent protein (GFP) with leucine zipper domains (Zip)) caspase-3/-7 reporter, a genetically engineered, caspase-activatable fluorescent biosensor based on a split-GFP architecture. In this design, the GFP molecule is divided into two parts: β-strands 1–10 and the eleventh β-strand [13], which are tethered via a flexible linker containing a caspase-3/-7-specific DEVD cleavage motif. Under basal conditions, the forced proximity of the β-strands prevents proper folding and chromophore maturation, resulting in minimal background fluorescence. Upon activation of caspase-3 or caspase-7 during apoptosis, cleavage at the DEVD site separates the β-strands, allowing spontaneous refolding into the native β-barrel structure of GFP [11, 14, 15]. This structural reassembly leads to efficient chromophore formation and rapid fluorescence recovery [16], providing a highly specific, irreversible, and time-accumulating signal for caspase activation. The ZipGFP system thus offers substantial advantages over conventional single-fluorophore or fluorescence resonance energy transfer (FRET)-based caspase reporters by minimizing background noise, enhancing signal stability, and enabling persistent marking of apoptotic events at the single-cell level [11]. Furthermore, the self-assembling properties of the split-GFP fragments eliminate the need for external cofactors or additional enzymatic reactions, making the system particularly well-suited for long-term imaging studies in both 2D monolayers and complex 3D culture environments, including patient-derived organoids. By constitutively co-expressing mCherry, the system provides internal normalization for cell presence.

Moreover, we sought to investigate two emerging facets of apoptosis with critical biological and therapeutic relevance: First, apoptosis-induced proliferation (AIP), a compensatory process wherein apoptotic cells actively stimulate the proliferation of neighbouring surviving cells through the release of mitogenic factors such as epidermal growth factors (EGF) and interleukin-6 (IL-6) [17, 18]. AIP is increasingly recognized as a driver of tumour repopulation following cytotoxic therapies, contributing to therapy resistance, tumour recurrence, and metastatic dissemination [18, 19]. Thus, real-time detection of AIP dynamics provides essential insight into tumour biology, the therapy response of a heterogeneous tumour cell population, and treatment failure mechanisms. Second, ICD, a regulated form of cell death capable of initiating an adaptive immune response against dying cells [9]. A defining feature of ICD is the pre-apoptotic exposure of CALR [8]. CALR exposure precedes phosphatidylserine externalization and is both necessary and sufficient to drive efficient engulfment and cross-presentation of tumour antigens, leading to cytotoxic T-cell activation [7]. Among ICD markers, CALR is uniquely positioned as an early, mechanistically central event directly linked to immunogenicity, making it the most reliable biomarker for functional ICD assessment [20].

Here, we describe the development, validation, and application of a reporter system capable of simultaneously tracking caspase-3/-7 activation and AIP or CALR exposure in cell culture models. We demonstrate its utility in monitoring apoptotic cell death kinetics, capturing apoptosis-induced proliferation, and identifying immunogenic features of cell death by simultaneous quantification of CALR exposure *via* flow cytometry. This integrated platform offers a high-content, scalable approach for mechanistic dissection of cell death modalities, therapeutic evaluation, and the study of cancer-immune interactions.

## Results

### Generation and validation of stable caspase-3/-7 reporter cells

**Fig. 1:**
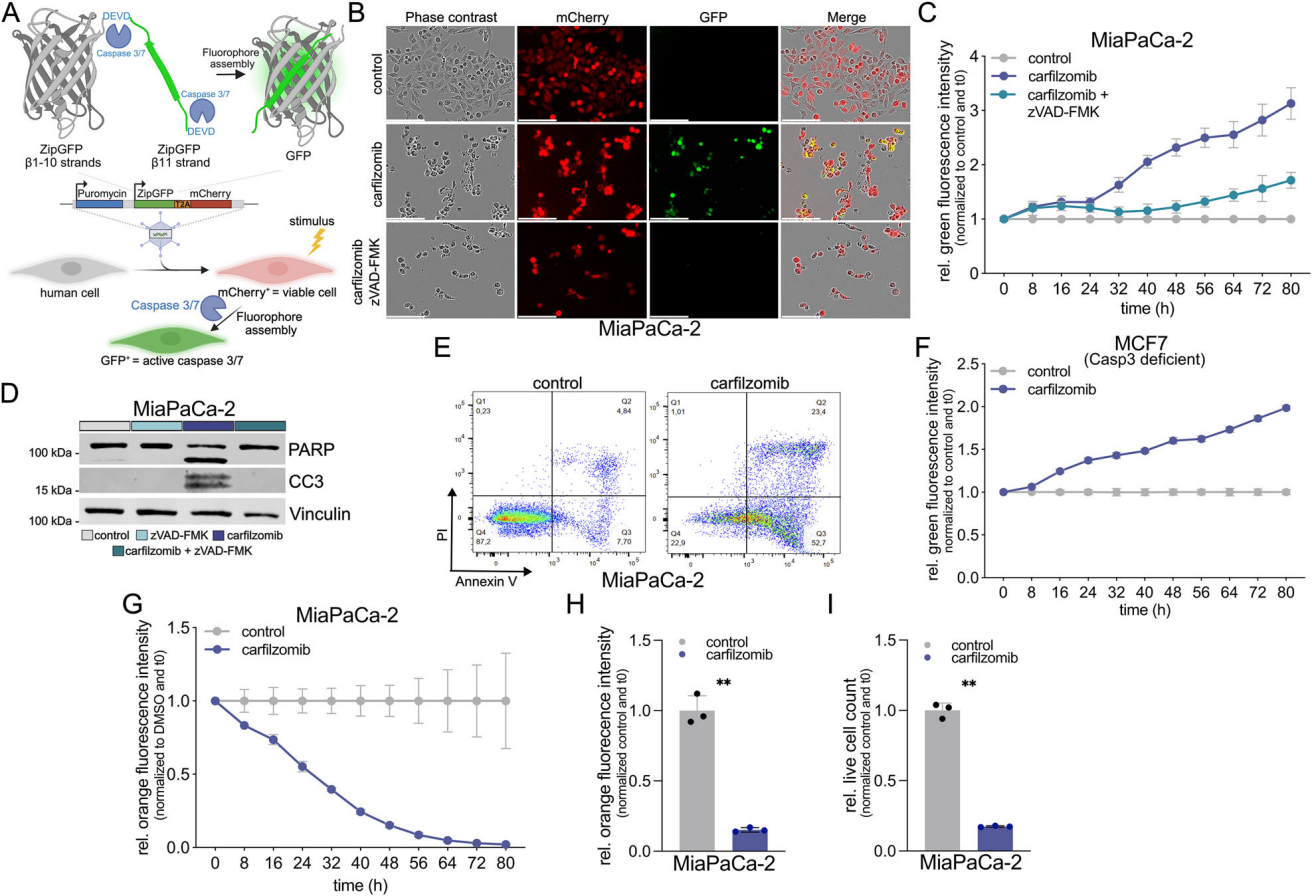
Generation and assessment of a ZipGFP-based caspase-3/-7 reporter. **A** Live-cell fluorescence imaging of reporter cells under basal and apoptotic conditions. A caspase-3/-7 reporter construct was introduced into human cells via lentiviral transduction to achieve stable expression. mCherry is constitutively expressed as a marker for successful transduction and cell presence, while GFP is assembled following caspase-3/-7-dependent cleavage of the DEVD motif, providing a real-time readout of apoptosis at the single-cell level. **B** Representative images of MiaPaCa-2 caspase reporter cells treated for 48 h with carfilzomib (50 nM) or DMSO (vehicle control), with or without zVAD-FMK (50 µM), showing phase-contrast, mCherry, and GFP channels. Scale bar: 150 µm. **C** Quantification of GFP fluorescence intensity over 80 h, with measurements taken every 8 h. Displayed is the relative green fluorescence intensity of MiaPaCa-2 caspase reporter cells treated with carfilzomib (50 nM), carfilzomib (50 nM) + zVAD-FMK (50 µM) or DMSO (vehicle control). Data represent mean ± SD of three independent experiments (*n* = 3). Statistical significance between treatment groups at each time point was assessed using two-way ANOVA with Šidák’s multiple comparisons test (t_80h_ *p* < 0.0001). **D**, **E** End-point apoptosis assessment by immunoblotting for cleaved PARP (*n* = 4) and cleaved caspase-3 (*n* = 1) (**D**) and by flow cytometry using Annexin V/PI staining (**E**) after 48 h of treatment with carfilzomib or DMSO as vehicle control. The results shown are representative of three independent experiments (*n* = 3). **F** Relative GFP fluorescence intensity in caspase-3 deficient MCF-7 reporter cells treated with carfilzomib, under the same imaging conditions as (**C**). Data represent mean ± SD of three independent experiments (*n* = 3). Statistical significance between treatment groups at each time point was assessed using two-way ANOVA with Sidak’s multiple comparisons test (t_80h_ *p* < 0.0001). **G**–**I** Quantification of mCherry fluorescence intensity over 80 h after treatment with carfilzomib or DMSO, with measurements taken every 8 h (**G**) mCherry intensity at 48 h (**H**) and corresponding live cell counts (**I**) obtained using the IncuCyte^®^ AI-based health monitoring module. Data represent mean ± SD of three independent experiments (*n* = 3). Statistical significance for (**H**) and (**I**) was assessed by paired *t*-test (**p < 0.01, ****p < 0.0001).

To enable real-time monitoring of apoptosis, we generated stable cell lines expressing a lentiviral-delivered caspase-3/-7 reporter carrying ZipGFP alongside a constitutive mCherry marker (Fig. 1A). In this system, the DEVD motif embedded in the ZipGFP construct enables fluorescence reconstitution specifically upon caspase-3/-7 activation (Table 1).

**Table 1:** Caspase specificity and DEVD cleavage summary.

Caspase	Cleaves DEVD	Preferred Motif	Function / Role
Caspase-1	−	WEHD, YVHD, FESD	Inflammatory (IL-1β activation)
Caspase-2	+	VDVAD, XDEVD	Apoptotic / stress response
Caspase-3	+++	DEVD	Executioner (apoptosis)
Caspase-4	−	LEVD, WEHD-like	Inflammatory (LPS sensing)
Caspase-5	−	LEVD, WEHD-like	Inflammatory (LPS sensing)
Caspase-6	++	VQVD,VEVD	Executioner (apoptosis, neurodegeneration)
Caspase-7	+++	DEVD	Executioner (apoptosis)
Caspase-8	++	LETD, XEXD	Initiator (extrinsic pathway)
Caspase-9	+	LEHD, WEHD	Initiator (intrinsic pathway)
Caspase-10	+	LEHD	Initiator (extrinsic pathway, similar to CASP8)
Caspase-11	−	WEHD-like	Inflammatory (non-canonical inflammasome in mice)
Caspase-12	−	Unclear	Controversial (mainly in rodents)
Caspase-13	n.a.	n.a.	Not in humans (bovine caspase)
Caspase-14	−	VEHD, VSQD/HSED	Skin differentiation (not apoptotic)

Cleaves DEVD: - no + very weak ++ weak +++ strong.

Following treatment with the apoptosis-inducing proteasome inhibitor carfilzomib, reporter cells exhibited a significant increase in GFP fluorescence, as visualized by time-lapse live-cell imaging (Fig. 1B, C). Quantitative analysis over 80 hours demonstrated a robust and time-dependent induction of GFP signal in treated cells compared to DMSO controls (Fig. 1C).

Co-treatment with the pan-caspase inhibitor zVAD-FMK [21] abrogated the GFP signal, confirming that reporter activation is caspase-dependent (Fig. 1B, C). Western blot analysis corroborated these findings by revealing increased levels of cleaved PARP and cleaved caspase-3 following carfilzomib treatment (Fig. 1D, Supplementary Fig. 1A; original blots in ‘Full blot scans’). Flow cytometric Annexin V/PI staining further validated the induction of apoptosis (Fig. 1E). To further validate the functionality and specificity of the caspase-3/-7 reporter system, we performed extended time-lapse imaging over 120 hours following treatment with oxaliplatin alone or in combination with zVAD-FMK. Consistent with caspase-3/-7 activation, oxaliplatin treatment induced a progressive increase in GFP fluorescence, whereas co-treatment with zVAD-FMK effectively suppressed reporter activation, confirming the caspase specificity of the signal (Supplementary Fig. 1B). To address caspase specificity in more detail, we applied the caspase-3/-7 reporter system to MCF-7 cells, which are caspase-3 deficient [22]. Despite the absence of caspase-3, MCF-7 reporter cells still exhibited a significant GFP signal upon carfilzomib treatment, indicating that caspase-7-mediated DEVD cleavage is sufficient for reporter activation (Fig. 1F).

In our system, mCherry is stably expressed, providing a persistent marker of successful transduction and cell presence. However, due to the inherent long half-life of the mCherry protein (approximately 24–30 h in mammalian cells), mCherry fluorescence is not suitable for direct, real-time assessment of cell viability following acute cell death [23]. Thus, mCherry primarily serves as a normalization control for fluorescence-based assays rather than a dynamic indicator of cell viability. However, automated analysis of live-cell counts using mCherry and the IncuCyte® AI Cell Health Module revealed a corresponding decrease in viable cell numbers, aligning with the observed GFP activation (Fig. 1G–I).

These results collectively validate the specificity, sensitivity, and robustness of the dual reporter system for real-time apoptosis tracking and limited viability assessment via mCherry.

### Application of the caspase-3/-7 reporter in 3D spheroid and organoid models

**Fig. 2:**
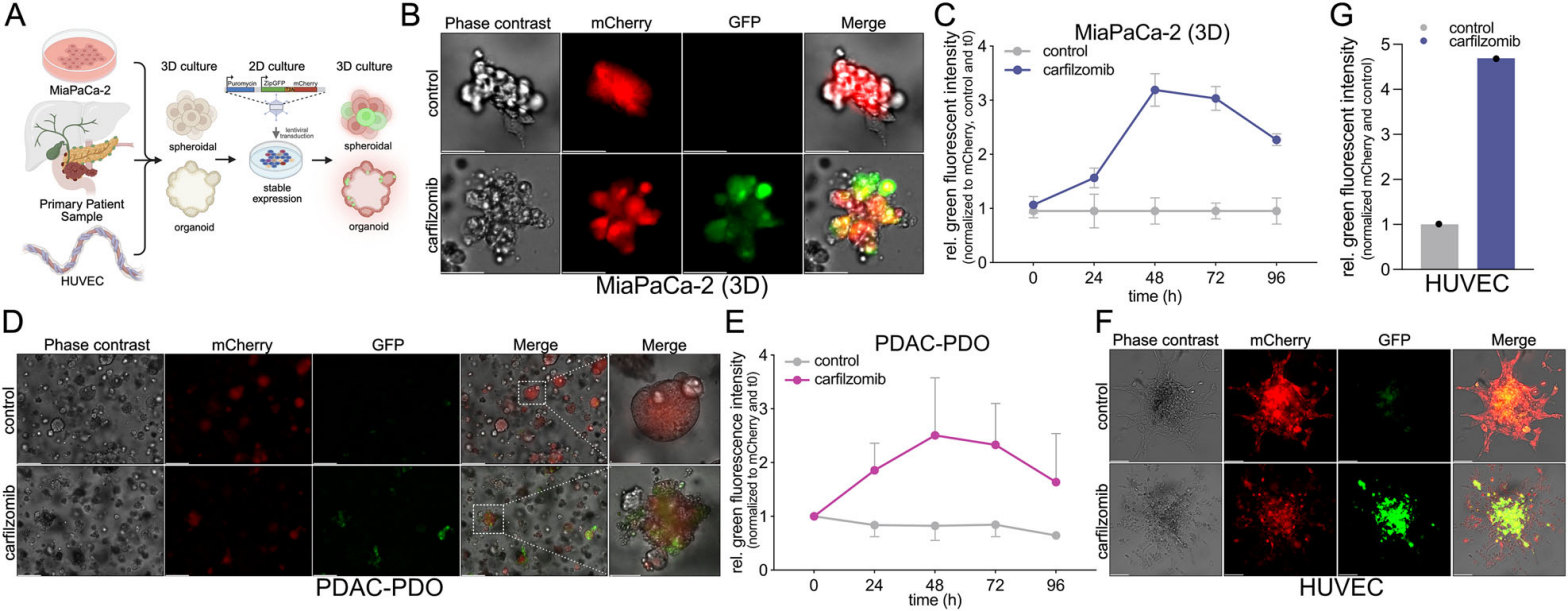
Spatiotemporal monitoring of caspase-3/-7 activity in 3D spheroid and organoid models. **A** Schematic overview of the experimental workflow for real-time monitoring of caspase activity in 3D culture systems using stable caspase-3/-7 reporter cells derived from the established cell culture model MiaPaCa-2 growing in spheroidal structures, primary patient derived organoid (PDO) and human umbilical vein endothelial cells (HUVECs) growing in spheroids. **B**, **C** MiaPaCa-2 caspase reporter cells embedded in Cultrex™, forming grape-like 3D spheroidal structures. **B** Representative phase-contrast and fluorescence images at 48 h post-treatment with Carfilzomib (50 nM) or DMSO as vehicle control. Scale bar: 50 µm. **C** Quantification of GFP fluorescence over time, normalized to baseline (t_0h_), DMSO control, and mCherry signal to account for cell number. Data represent mean ± SD of three independent experiments (*n* = 3). Statistical significance between treatment groups at each time point was assessed using two-way ANOVA with Šidák’s multiple comparisons test (t_96h_ p < 0.0001). **D**, **E** Patient-derived organoid (PDAC-PDO) cultures embedded in Cultrex™ and treated with carfilzomib (50 nM) or DMSO. **D** Representative images at 48 h showing phase-contrast, mCherry, and GFP channels. Scale bars: 100 µm (panels 1–4); 30 µm (panel 5). **E** Quantification of GFP fluorescence intensity over 96 h after treatment with carfilzomib or DMSO, with measurements taken every 24 h. Data represent mean +SD (carfilzomib) and -SD (DMSO) of four independent experiments (*n* = 4). Statistical significance between treatment groups at each time point was assessed using two-way ANOVA with Sidak’s multiple comparisons test (t_24h_ *p* < 0.05, t_48h_ *p* < 0.001, t_72h_ *p* < 0.01, t_96h_ not significant). **F**, **G** Primary HUVEC spheroids, generated by the hanging drop method, embedded in hydrogel and treated with carfilzomib (100 nM) or DMSO. **F** Representative images at 24 h post-treatment. Scale bar: 100 µm. **G** Quantification of relative GFP fluorescence intensities after 24 h treatment with carfilzomib or DMSO (*n* = 1). All fluorescence quantifications (**C**, **E**, **G**) were normalized to baseline (t_0h_), DMSO control, and mCherry signal.

To extend the utility of the caspase-3/-7 reporter system to more physiologically relevant models, we applied it to 3D cultures including endothelial spheroids and patient-derived pancreatic ductal adenocarcinoma (PDAC) organoids (Fig. 2A).

MiaPaCa-2 cell-derived spheroids embedded in Cultrex^TM^ exhibited a time-dependent increase in GFP signal following apoptosis induction (Fig. 2B, C). Importantly, fluorescence normalization to mCherry intensity ensured accurate interpretation of apoptosis independent of changes in cell viability. In patient-derived organoid (PDAC-PDO) cultures, localized GFP fluorescence was observed upon carfilzomib treatment, indicating the activation of apoptosis within heterogeneous organoid structures (Fig. 2D, E).

Additionally, in HUVEC-derived spheroids, fluorescence imaging demonstrated stable mCherry expression and a marked induction of GFP fluorescence following carfilzomib treatment, reflecting caspase activation in the 3D context (Fig. 2A, F). Quantification revealed an increase in GFP intensity relative to control (Fig. 2G). Together, these findings demonstrate that the caspase-3/-7 reporter system enables robust, real-time detection of apoptosis in complex 3D environments, including both engineered spheroids and clinically relevant PDO models.

### Real-time monitoring of apoptosis-induced proliferation

**Fig. 3:**
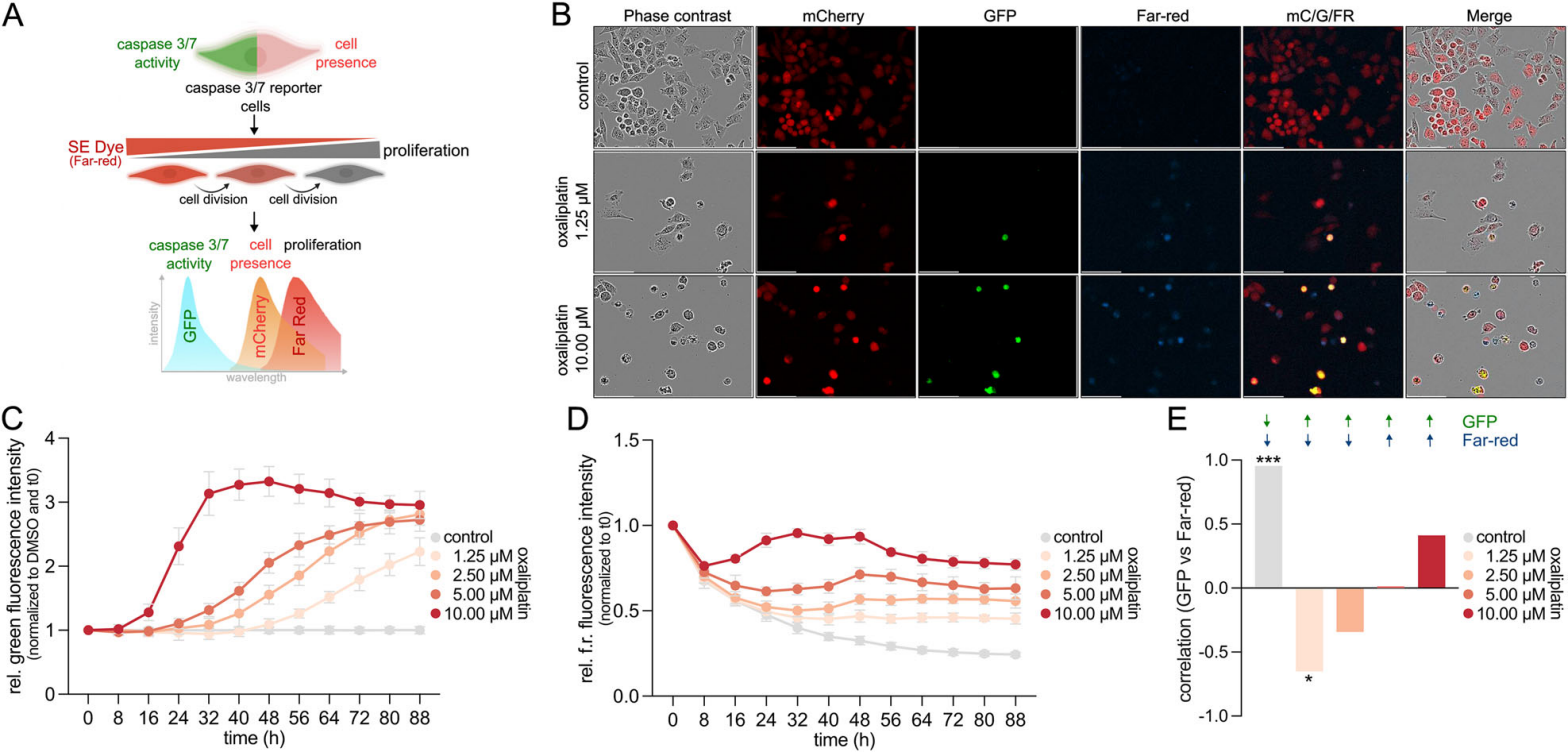
Real-time monitoring of apoptosis-induced proliferation. **A** Schematic overview of the triple-reporter strategy: dual caspase-3/-7 reporter combined with a far-red succinimidyl ester (SE) proliferation dye to simultaneously monitor viability, caspase activity, and proliferation dynamics. **B** Representative images of reporter cells treated with 1.25 µM or 10 µM oxaliplatin and vehicle control, showing mCherry, GFP, and far-red proliferation dye fluorescence. Scale bar: 100 µm. **C** Time-course of relative GFP fluorescence intensity normalized to t0 and vehicle control, demonstrating a dose-dependent increase in caspase activation across oxaliplatin concentrations (1.25; 2.5; 5; 10 µM). Data represent mean ± SD of three independent experiments (*n* = 3). **D** Time-course of relative far-red fluorescence intensity normalized to t_0h_, illustrating dye dilution as a proxy for cell proliferation after treatment with different oxaliplatin concentrations and vehicle control as described in (**C**). Data represent mean ± SD of three independent experiments (*n* = 3). **E** Correlation between GFP and far-red signals for each treatment condition, calculated across individual fields of view from (**C**) and (**D**) using Spearman’s rank correlation (*p < 0.05, ***p < 0.001).

Beyond apoptosis quantification, we explored the capacity of our platform to detect apoptosis-induced proliferation (AIP), a compensatory proliferative response occurring in neighboring cells following apoptotic injury. To achieve this, caspase-3/-7 reporter cells were labeled with a cell-permeable, far-red succinimidyl ester (SE) proliferation dye [24]. Upon cell division, the dye is diluted, allowing proliferation tracking alongside apoptosis monitoring (Fig. 3A). Following treatment with escalating concentrations of oxaliplatin, we observed a dose-dependent increase in GFP fluorescence (indicating caspase activation) alongside a decrease in far-red dye intensity (indicating cell proliferation) specifically in low doses (Fig. 3B–D). Spearman correlation analysis between GFP and far-red signals revealed a significant negative correlation at sublethal doses observed across individual fields of view (Fig. 3E), suggesting that regions with moderate apoptotic activity exhibited compensatory proliferation among surviving cells.

These findings highlight the capability of the reporter platform to simultaneously monitor apoptotic cell death, viability, and regenerative proliferative responses within the same system.

### Simultaneous detection of caspase activation and immunogenic cell death via calreticulin exposure

**Fig. 4:**
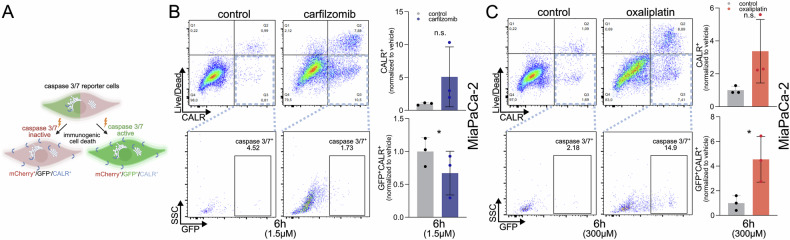
The caspase reporter system enables parallel quantification of immunogenic cell death and caspase activation. **A** Simultaneous flow cytometric quantification of calreticulin (CALR) surface exposure, alongside caspase-3/-7 activation, allows discrimination between different modes of cell death. **B**, **C** Simultaneous staining of surface CALR (using rabbit anti-CALR + anti-rabbit-PE) and cell viability (using Zombie Aqua) after treatment with 1,5 µM carfilzomib (**B**) or 300 µM oxaliplatin for 6 h (**C**) shows different levels of ICD (upper panels) and caspase induction (lower panels). Data represent mean ± SD of three independent experiments (*n* = 3). Statistical significance was assessed by paired two-tailed *t*-test (*p < 0.05; n.s.: not significant).

To dissect immunogenic features of cell death, we developed a dual-detection approach combining real-time caspase-3/-7 reporter imaging with flow cytometric measurement of calreticulin (CALR) exposure (Fig. 4A). CALR, an essential “eat-me” signal for dendritic cell activation, is externalized on the plasma membrane during ICD but not during non-immunogenic apoptosis.

Upon treatment with carfilzomib, MiaPaCa-2 caspase-3/-7 reporter cells surprisingly exhibited surface exposure of CALR, detected by flow cytometry using anti-CALR antibodies (Fig. 4B). Notably, these CALR⁺ cells did not show activation of caspase-3/-7, indicating a caspase-independent form of immunogenic cell death (ICD). This observation was unexpected, as carfilzomib-induced ICD is context-dependent and has been primarily demonstrated in multiple myeloma [25]. In contrast, treatment with oxaliplatin, a well-established ICD inducer, also led to CALR surface exposure, albeit to a lesser extent than carfilzomib (Fig. 4C). Importantly, in oxaliplatin-treated cells, a distinct subpopulation of CALR⁺ cells also exhibited caspase-3/-7 activation, suggesting the coexistence of both caspase-dependent and caspase-independent ICD pathways (Fig. 4C). These findings underscore the utility of the caspase-3/-7 reporter system in combination with CALR detection to distinguish between different forms of ICD. Simultaneous live/dead assessment enabled the exclusion of necrotic artifacts.

These results demonstrate that the caspase-3/-7 reporter system, when coupled with CALR detection, enables discrimination between immunogenic and non-immunogenic forms of cell death, providing a versatile platform for evaluating cell death mechanisms and potential immune activation.

## Discussion

In this study, we developed and validated a stable, dual-fluorescent reporter system combining ZipGFP - a caspase-3/-7-sensitive biosensor - with constitutive mCherry expression. This platform enables real-time visualization of apoptosis, apoptosis-induced proliferation, and assessment of immunogenic cell death (ICD) in both 2D and 3D culture models, including endothelial spheroids and patient-derived organoids (PDOs).

The caspase-3/-7 reporter system demonstrated high specificity for executioner caspase activation, as evidenced by GFP fluorescence induction following apoptotic stimuli and its abrogation by pan-caspase inhibition. Notably, the reporter maintained functionality in caspase-3-deficient MCF-7 cells, indicating that caspase-7-mediated cleavage suffices for activation [3, 11, 25]. Extending this platform to 3D models addresses a significant limitation of traditional endpoint assays, which often struggle with reagent penetration and photobleaching artifacts in spheroids and organoids [26]. Our system provides dynamic, spatially resolved tracking of apoptosis within multicellular structures without additional staining, offering a physiologically relevant readout.

A key innovation of our study is the simultaneous monitoring of apoptosis-induced proliferation (AIP). AIP is increasingly recognized as a critical mechanism by which tumours evade cytotoxic therapies. Apoptotic cells can release mitogenic signals, such as EGF, stimulating proliferation in neighbouring cells and contributing to tumour repopulation [27, 28]. Recent studies have highlighted the role of caspase activity in promoting regenerative proliferation even following necrosis, underscoring the complex interplay between cell death and tissue regeneration [29, 30]. By coupling the caspase-3/-7 reporter with a cell proliferation dye, we observed a significant inverse correlation between apoptotic activity and proliferation, providing direct evidence of compensatory proliferation dynamics following therapy.

Our platform also facilitates the assessment of ICD, a form of regulated cell death that primes adaptive immune responses. Among ICD hallmarks, calreticulin (CALR) surface exposure is a pivotal early event, facilitating efficient recognition and engulfment by dendritic cells [8]. Recent research has further emphasized CALR’s role in activating dendritic cells and promoting cross-priming of cytotoxic T cells, particularly in the context of cancer immunotherapy [10, 31]. By integrating caspase-3/-7 imaging with flow cytometric measurement of CALR exposure, we demonstrated the system’s capability to distinguish between immunogenic and non-immunogenic cell death modalities.

The versatility of the caspase-3/-7 reporter system extends beyond apoptosis and ICD monitoring. Its applicability in complex 3D models and compatibility with high-content imaging and flow cytometry make it a valuable tool for drug screening and mechanistic studies. Future directions could involve incorporating sensors for other regulated cell death modalities, such as pyroptosis, ferroptosis, or PANoptosis [6, 32, 33], further expanding the system’s utility. Additionally, adapting the platform for in vivo imaging could translate real-time cell death monitoring into preclinical models, enhancing our understanding of therapeutic responses in a physiological context.

Together, the stable caspase-3/-7 reporter system we present offers a robust, scalable platform for high-resolution, real-time dissection of apoptosis dynamics, apoptosis-induced proliferation, and immunogenic cell death in different cell culture models. By integrating spatiotemporal tracking of caspase activity and surface calreticulin exposure, this system significantly advances the mechanistic study of regulated cell death and provides a versatile tool for therapeutic discovery, resistance monitoring, and immuno-oncology research.

## Methods

### Cell lines

Human pancreatic cancer MiaPaCa-2 (RRID: CVCL_0428), breast cancer MCF-7 (RRID: CVCL_0031), and embryonic kidney HEK293FT (RRID: CVCL_6911) cell lines were authenticated by short tandem repeat (STR) profiling. Cell lines were routinely tested for mycoplasma contamination using the MycoAlert™ Mycoplasma Detection Kit (Lonza, LT-07318) according to the manufacturer’s instructions. Cells were maintained under standard conditions in a humidified incubator at 37 °C with 5% CO₂. MiaPaCa-2 and HEK293FT cells were cultured in high-glucose Dulbecco’s Modified Eagle Medium (DMEM; Sigma-Aldrich, D6429) supplemented with 10% fetal bovine serum (FBS; Eurobio, CVFSVF00-01). MCF-7 cells were maintained in high-glucose RPMI 1640 medium (Sigma-Aldrich, R8758) supplemented with 10% FBS.

### Primary cell cultures

Primary Human Umbilical Vein Endothelial Cells (HUVECs) were cultured in a 1:1 mixture of endothelial growth medium (EGM-2, Lonza) and RPMI 1640 (Sigma-Aldrich, R8758). Plates were pre-coated with 0.1% gelatin (Sigma-Aldrich, G9391) to enhance cell adhesion. HUVECs were maintained at 37 °C in a humidified 5% CO₂ atmosphere and passaged at approximately 80% confluency, using only early passages to preserve endothelial characteristics. Patient-derived pancreatic ductal adenocarcinoma cells (PDAC-PDO) established as recently described [34] were cultured in RPMI 1640 supplemented with 20% FBS and maintained under identical incubator conditions.

### Spheroid formation Assay

For spheroids formation 5 × 10^5^ HUVECs were mixed with 10 ml of supplemented Endothelial Cell Growth Medium 2 (ECGM2, #22011, PromCell, Heidelberg, Germany) supplemented with 2% v/w sterile methylcellulose (Sigma-Aldrich, #M0555, St. Louis, USA). 25 µl of the cell suspension were plated on 10 cm squared plates (Sigma-Aldrich, #CLS431272-16A), turned upside down (hanging drops) for overnight incubation (approx. 16 h). Next day, spheroids were gently washed and collected into a 50 ml conical tube with approximately 10 ml of PBS (Gibco) supplemented with 10% fetal bovine serum (FBS) (PAN Biotech GmbH, #P30-3306, Aidenbach, Germany). After 5 min centrifugation at 300 g, RT, no brakes, ECGM2 medium (PromCell) supplemented with 5 mg/ml fibrinogen (Sigma-Aldrich, #F3879) was gently overlayed on the pellet and mixed. For solidification 0.6 Units (approx. 6 µl/ml) Thrombin (Sigma-Aldrich, #T4648) was added and again gently mixed. 500 µl of the mixture-containing spheroids was plated into 24-well-plates (SARSTEDT, #83.3922.005, Hildesheim, Germany).

### Cloning and stable cell line generation

For cloning of the lentiviral expression vector the DEVD-ZipGFP-T2A-mCherry sequence was synthesized as a gBlock (Integrated DNA Technologies) based on the caspase-3/-7-sensitive biosensor design described by To et al., 2016 [11]. For Gateway-based cloning, attB1 and attB2 recombination sites were incorporated at the 5’ and 3’ ends of the gBlock, respectively. The synthesized fragment was cloned into a pENTR/D-TOPO entry vector (Thermo Fisher Scientific) using standard BP recombination reactions. Subsequently, an LR Clonase II (Thermo Fisher Scientific) reaction was performed to recombine the insert into the pLEX307 destination vector (Addgene plasmid #41392), which contains a puromycin resistance cassette for selection. Successful cloning was confirmed by restriction digest analysis and Sanger sequencing. Stable expression of the caspase-3/-7 reporter was achieved through lentiviral transduction. Briefly, HEK293FT cells were seeded at a density of 7 × 10⁵ cells per well in six-well plates and co-transfected with the caspase-3/-7 plasmid, packaging plasmid psPAX2 (Addgene #12260), and envelope plasmid pMD2.G (Addgene #12259) using Lipofectamine 3000 (Thermo Fisher Scientific, L3000008) according to the manufacturer’s instructions. After 48 h, viral supernatants were harvested, filtered through 0.45μm membranes, and used to transduce MiaPaCa-2, MCF-7, PDAC-PDOs, and HUVECs in the presence of 8 μg/mL polybrene (Sigma-Aldrich, H9268). Forty-eight hours post-infection, cells were selected with 1 μg/mL puromycin (Carl Roth, T830.3) for seven days to establish stable reporter cell populations.

### 3D cell culture

For 3D culture experiments, MiaPaCa-2 and PDAC-PDO caspase-3/-7 reporter cells were embedded in Cultrex™ Reduced Growth Factor Basement Membrane Extract, Type 2, Select (R&D Systems, 3536-005-02). A total of 7.5 × 10^3^ or 1 × 10^4^ cells per 50 μL Cultrex™ dome were plated into pre-warmed 24-well plates and allowed to solidify at 37 °C for 30 min. Domes were then overlaid with 500 μL human complete feeding medium, consisting of advanced DMEM/F12 supplemented with 10% R-spondin- and Noggin-conditioned medium, B27 supplement, HEPES, GlutaMAX, nicotinamide, N-acetylcysteine, and additional growth factors. Media was refreshed every two days. After three to seven days of growth, depending on the model, cells were treated with carfilzomib (Selleck Chemicals, S2853) at indicated concentrations. Live imaging was performed daily over 96 hours using Olympus IX83 inverted microscope equipped with the cellSens Dimension software (version 3.2; Olympus, Tokyo, Japan) for image acquisition. Z-stack images were captured using standard settings, with exposure times kept constant across conditions, and were subsequently analyzed using ImageJ (Fiji distribution). Fluorescent signals were detected using the FITC channel (GFP) and the Cy3 channel (mCherry). All imaging parameters, including laser intensity and Z-step size, were maintained consistently throughout the experiment to ensure reproducibility.

### Live-cell imaging and caspase activity assay

To monitor caspase activation and cell proliferation simultaneously, caspase-3/-7 reporter cells were seeded into 96-well flat-bottom plates (Sarstedt, 83.3924) at optimized densities and treated with 50 nM carfilzomib or various concentrations of oxaliplatin. Cells were also labeled with CellTrace™ Far Red (Thermo Fisher Scientific, C34564) prior to treatment for proliferation tracking according to the manufacturer’s instructions. Live-cell imaging was conducted every 8 hs for up to 120 h using the IncuCyte® S5 Live-Cell Analysis System (Sartorius), capturing fluorescence in the green (GFP), orange (mCherry), and far-red channels. Quantification of fluorescence intensity was performed using Sartorius IncuCyte® image analysis software (version 2024 A and 2024B), and automated live-cell counts were performed using the integrated IncuCyte® AI Cell Health Analysis Module.

### Western blotting

Cells were lysed in ice-cold RIPA buffer (Thermo Fisher Scientific), supplemented with protease and phosphatase inhibitors (Roche). Lysates were clarified by centrifugation at 14,000 × g for 15 min at 4 °C, and protein concentrations were determined using the Roti®-Quant Bradford assay (Carl Roth). Equal amounts of protein were mixed with Laemmli buffer, denatured at 95 °C for 5 min, and separated on 10–20% Tris-Glycine SDS-PAGE gels. Total protein staining was achieved by incorporating 0.5% trichloroacetic acid (TCA) into the separating gel. Following transfer onto nitrocellulose membranes, protein loading was verified via UV activation imaging (Bio-Rad ChemiDoc). Membranes were blocked with 5% non-fat dry milk in TBS-T and probed with primary antibodies (PARP (Cell Signaling Technologies, 9532S, RRID: AB_659884), cleaved caspase-3 (Cell Signaling Technologies, 9664S, RRID: AB_2070042) and vinculin (Santa Cruz Biotechnology, sc-73614, RRID: AB_1131294)) overnight at 4 °C, followed by incubation with fluorescent (Alexa Fluor® 546 goat anti-rabbit IgG (H + L) (A11035, Invitrogen, RRID: AB_2534077), and Alexa Fluor® Plus 488 goat anti-mouse IgG (H + L) (A32723, Invitrogen, RRID: AB_2866489)), or HRP-conjugated (goat anti-rabbit IgG (H + L) (R1364HRP, Origene, RRID: AB_2622246)), secondary antibodies. Signal detection was performed using either the LI-COR Odyssey or Bio-Rad imaging systems. Densiometric analysis was performed using ImageJ (Fiji distribution). Original, uncropped blot scans provided in the supplemental materials.

### Flow cytometry

Apoptotic and immunogenic cell death markers were assessed by flow cytometry using a FACSCelesta cytometer (Beckman Coulter). Cells were stained with Annexin V-FITC (BD, #556419) and propidium iodide (PI) (BioLegend, #421301) according to the manufacturer’s protocol to evaluate early and late apoptosis. For CALR detection, cells were stained with rabbit anti-CALR (abcam, AB2907) followed by secondary staining using donkey-anti-rabbit-PE (BioLegend, #406421) antibodies, enabling assessment of calreticulin surface exposure. Viability staining was performed using Zombie Aqua Fixable Viability Dye (BioLegend, #423101) according to the manufacturer’s instructions. Simultaneous acquisition of mCherry, GFP, and PE signals allowed for multiparametric analysis of viability, caspase activation, and immunogenic markers. Appropriate compensation and gating strategies were applied to correct for spectral overlap, and data were analyzed using FlowJo 10.8 software.

### Statistical analysis

All quantitative data are presented as mean ± standard deviation (SD) from indicated number (n) of independent biological replicates, unless otherwise specified. Statistical analyses were performed using GraphPad Prism 10.2.3 (GraphPad Software, San Diego, CA, USA, RRID: SCR_002798). Differences between the two groups were assessed using paired or unpaired two-tailed Student’s t-tests, depending on experimental design. Significance between treatment groups at different time points were evaluated by two-way ANOVA followed by Šidák’s multiple comparisons test. Correlations between apoptosis and proliferation metrics were evaluated using Spearman’s rank correlation coefficient. *P*-values less than 0.05 were considered statistically significant, and significance levels are denoted in figure legends where applicable (****p < 0.0001, ***p < 0.001, **p < 0.01, *p < 0.05).

### Use of Large Language Model

Parts of the manuscript, including language refinement, reorganization, and assistance in scientific phrasing, were supported using OpenAI’s ChatGPT (April 2025 version). The authors confirm that all intellectual content, experimental design, data interpretation, and scientific conclusions were solely generated and reviewed by the authors.

## Supplementary information

Supplementary Figure 1

Supplementary Figure Legends

Full blot scans

## Data Availability

The data that support the findings of this study are available from the corresponding author upon reasonable request.
