# Supplementary figures and images for: Integrated real-time imaging of executioner caspase dynamics, apoptosis-induced proliferation, and immunogenic cell death using a stable fluorescent reporter platform
Source: Cell Death Discov. 2025 Aug 6;11:368. doi: 10.1038/s41420-025-02662-y (PMC12328661; doi:10.1038/s41420-025-02662-y)

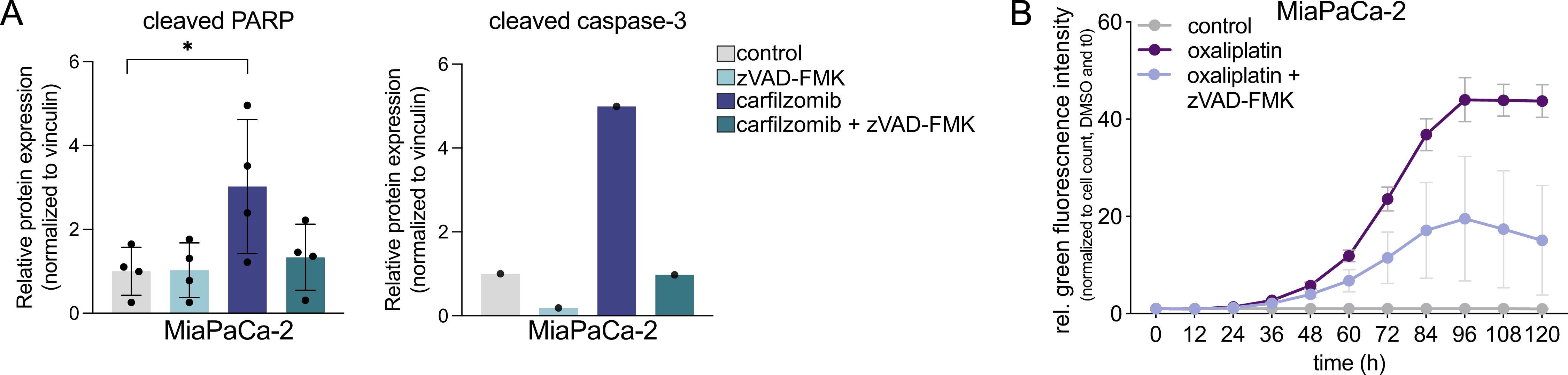

Supplement: Supplementary file 1 — Supplementary Figure 1 [file 41420_2025_2662_MOESM1_ESM.png]

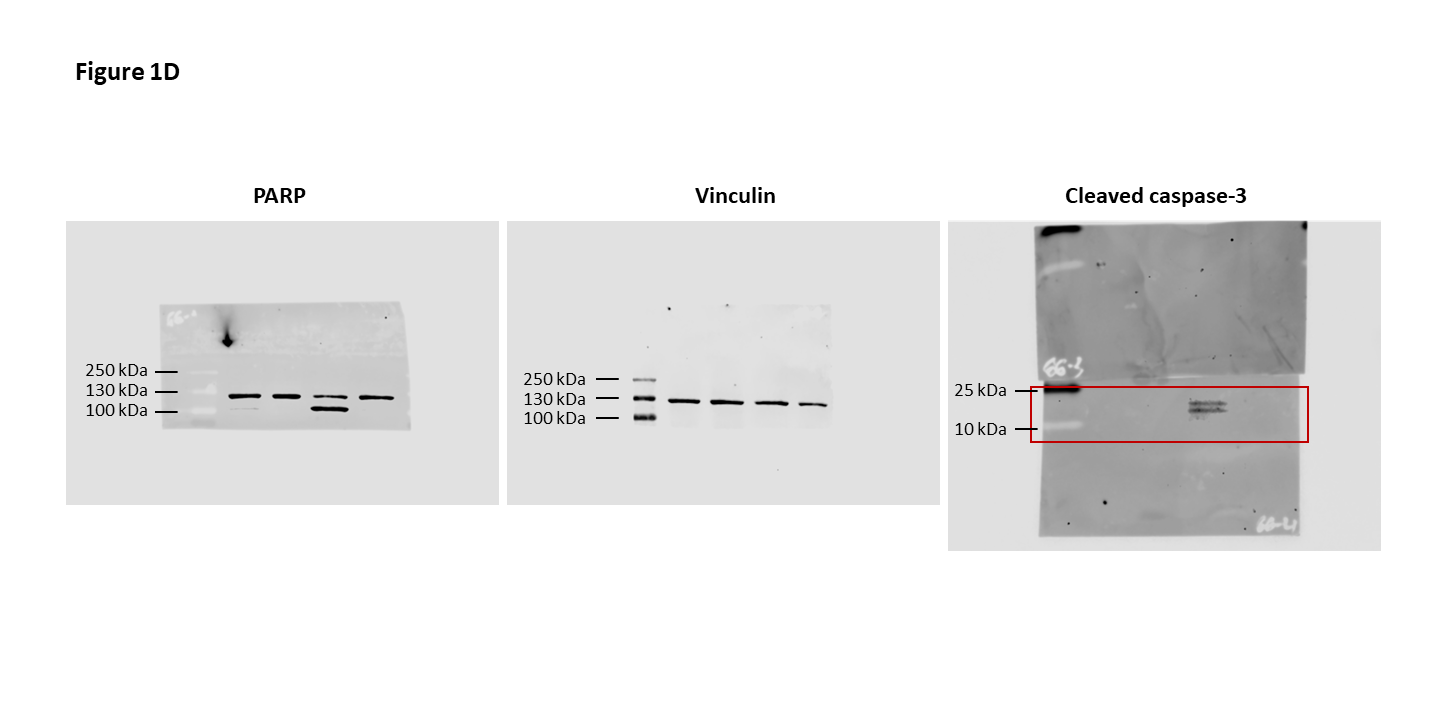

Supplement: Supplementary file 3 — Full blot scans [file 41420_2025_2662_MOESM3_ESM.tif]
