# Supplementary material for: Integrated real-time imaging of executioner caspase dynamics, apoptosis-induced proliferation, and immunogenic cell death using a stable fluorescent reporter platform
Source: Cell Death Discov. 2025 Aug 6;11:368. doi: 10.1038/s41420-025-02662-y (PMC12328661; doi:10.1038/s41420-025-02662-y)
Supplement: Supplementary file 2 — Supplementary Figure Legends [file 41420_2025_2662_MOESM2_ESM.docx]

**Supplementary Figure 1.**

(A) Densitometry of cleaved PARP and cleaved caspase-3 immunoblots.

Immunoblot quantification was performed by densitometric analysis using Fiji (ImageJ). Band intensities were normalized to the housekeeping protein vinculin, and results are presented as fold change relative to the control condition. Statistical test has been performed by an ordinary one-way ANOVA with Šidák’s multiple comparison test; * indicates a p-value < 0.05.

(B) Oxaliplatin induces caspase-3/-7 activity in MiaPaCa-2 cells.

Quantification of GFP fluorescence intensity over 120 h, with measurements taken every 12 h. Displayed is the relative green fluorescence intensity of MiaPaCa-2 caspase-reporter cells treated oxaliplatin (10µM), oxaliplatin (10µM) + zVAD-FMK (50µM) or DMSO (vehicle control). Data represent mean ± SD of **three independent experiments** (n = 3).
